# BEST: Better Equipped to Start Treatment

**DOI:** 10.1186/1758-2652-13-S4-P166

**Published:** 2010-11-08

**Authors:** J Bennett

**Affiliations:** 1 Co-Chair BEST Advisory Board, London, UK

## Introduction

BEST was developed by a board of key stakeholders of European community group representatives, People Living with HIV (PLWHIV), researchers, clinicians, nurses and medical organisations, representing 6 European countries, (UK, Germany, France, Italy, Portugal and Spain). BEST focuses on empowering PLWHIV to make decisions about starting Antiretroviral Therapy (ART) as per current EACS Guidelines. It aims to educate tested, but not treated PLWHIV on the benefits of starting ART according to current guidelines to improve their long-term health and quality of life.

## Rationale

In Europe many people start ART with a CD4 count lower than current guidelines recommend, with associated increased morbidity and mortality [1]. Lack of understanding of guidelines, and ineffective communication between PLWHIV and their healthcare professionals (HCPs) may delay the commencement of ART in PLWHIV.

## Programme development

The programme was developed by an Advisory Board, and Review Committee of HCPs and community members, with material content at the Boards' discretion. It is organised and funded by Bristol-Myers Squibb and Gilead Sciences.

## Materials

The toolkit contains 5 presentation sections to facilitate discussion around; Preparing and Readiness to Start ART: When to Start Treatment:

The Rationale; Reasons to start at a CD4 of 350 or above; Building the Best Relationship with Your HIV Clinic and HIV and ART: Questions and Answers. A 6th section, 'Running Workshops' provides information on running workshops, facilitating audience participation and evaluation. It is intended for use by advocacy workers and/or healthcare workers.

## Progress to date

BEST was launched at EACS 2009 to a group of mainly community advocacy workers from 6 European countries. Evaluation showed that the materials and workshop format were well received and addressed an unmet need in the countries represented. BEST was rolled out locally in the 1st half of 2010 in 6 pilot countries, where it has had considerable success. Feedback and evaluation from the national rollout will be provided in detail in this poster presentation.

## Future plans

We hope BEST will result in more PLWHIV starting ART at the recommended optimum time, possibly determined by prospective surveillance or audit at a local level. Evaluation systems will enable the BEST Advisory Board to respond to the changing needs of users; and to develop appropriate new materials. Regular updates to BEST are planned every 6 months.
